# Electroconvulsive therapy vs Esketamine among patients with Major Depressive Episode

**DOI:** 10.1192/j.eurpsy.2023.1771

**Published:** 2023-07-19

**Authors:** M. D. R. D. R. F. D. A. Basto, O. Nombora, L. Santa Marinha, J. L. Simães, A. Horta

**Affiliations:** Psychiatry and Mental Health, Centro Hospitalar de Vila Nova de Gaia e Espinho, Vila Nova de Gaia, Portugal

## Abstract

**Introduction:**

Major depressive disorder is one of the most common and disabling mental disorders. More than 30% of individuals do not achieve remission after several trials of antidepressants and treatment-resistant depression (TRD) is associated with premature mortality. Electroconvulsive therapy (ECT) is considered the gold-standard for TRD treatment,unfortunately it´s underused due to health care barriers and association with adverse cognitive impairment. So, scientists have sought to identify alternative treatments that approach ECT-equivalent efficacy. Trials with Ketamine and more recently with its S-enantiomer (Esketamine) has been made, revealing a rapid and robust antidepressant effect, emerging as an option for TRD treatment.

**Objectives:**

We we aim to conduct a qualitative review, comparing clinical efficacy, tolerability and acceptability between the use of Esketamine and ECT as a TRD treatment.

**Methods:**

We conduct a non-systematic review of recent evidence between the use of Esketamine and ECT as a TRD treatment, using PubMed/Medline database.

**Results:**

To compare clinical efficacy, tolerability and acceptability between the use of Esketamine and ECT as a TRD treatment we analyzed outcomes of interest. First,ECT was superior to Esketamine improving depressive symptoms. Comparing suicidal ideation and suicide attempts, most results were not statistically different. About cognition impairment, Esketamine performed better than ECT, particularizing attention, verbal memory, and executive functions; no differences were found for immediate memory or visual memory.About adverse effects Esketamine has less risk of headache and muscle pain, but higher reports of transient, dissociative or depersonalization symptoms, blurred vision, diplopia and nystagmus. An important consideration for clinicians is the comparative tolerability and safety of Esketamine vs ECT; as ECT involves a full dose of anesthesia,it is expected that Esketamine would be better tolerated and safer than ECT. But no study assessed the relative tolerability or acceptability of these different adverse effect profiles.The best strategy for relapse prevention appears to be continuing ECT, continuing pharmacotherapy, or using some combination of both; but Esketamine continuing treatment is effective too.

**Conclusions:**

ECT may be superior to Esketamine for improving depression severity in the acute phase, but long-term outcomes of these treatments are important to be considered. There are just two studies with long-term follow-up after the trial completed:one found no difference in depression severity during the 3-month follow-up, and the other reported that the remission rates were not different between groups by the 12-month follow-up period.Therefore,future research is needed to further optimize long-term treatment outcomes for both Esketamine and ECT to prevent relapse. Until then,treatment options should be individualized and patient-centered.

**Disclosure of Interest:**

None Declared

